# Walking-speed estimation using a single inertial measurement unit for the older adults

**DOI:** 10.1371/journal.pone.0227075

**Published:** 2019-12-26

**Authors:** Seonjeong Byun, Hyang Jun Lee, Ji Won Han, Jun Sung Kim, Euna Choi, Ki Woong Kim

**Affiliations:** 1 Department of Psychiatry, Seoul National University, College of Medicine, Seoul, Korea; 2 Department of Neuropsychiatry, National Medical Center, Seoul, Korea; 3 Department of Neuropsychiatry, Seoul National University Bundang Hospital, Seongnam, Korea; 4 Department of Brain and Cognitive Science, Seoul National University College of Natural Sciences, Seoul, Korea; 5 Korean National Institute of Dementia, Seongnam, Korea; Baylor College of Medicine, UNITED STATES

## Abstract

**Background:**

Although walking speed is associated with important clinical outcomes and designated as the sixth vital sign of the elderly, few walking-speed estimation algorithms using an inertial measurement unit (IMU) have been derived and tested in the older adults, especially in the elderly with slow speed. We aimed to develop a walking-speed estimation algorithm for older adults based on an IMU.

**Methods:**

We used data from 659 of 785 elderly enrolled from the cohort study. We measured gait using an IMU attached on the lower back while participants walked around a 28 m long round walkway thrice at comfortable paces. Best-fit linear regression models were developed using selected demographic, anthropometric, and IMU features to estimate the walking speed. The accuracy of the algorithm was verified using mean absolute error (MAE) and root mean square error (RMSE) in an independent validation set. Additionally, we verified concurrent validity with GAITRite using intraclass correlation coefficients (ICCs).

**Results:**

The proposed algorithm incorporates the age, sex, foot length, vertical displacement, cadence, and step-time variability obtained from an IMU sensor. It exhibited high estimation accuracy for the walking speed of the elderly and remarkable concurrent validity compared to the GAITRite (MAE = 4.70%, RMSE = 6.81 𝑐𝑚/𝑠, concurrent validity (ICC (3,1)) = 0.937). Moreover, it achieved high estimation accuracy even for slow walking by applying a slow-speed-specific regression model sequentially after estimation by a general regression model. The accuracy was higher than those obtained with models based on the human gait model with or without calibration to fit the population.

**Conclusions:**

The developed inertial-sensor-based walking-speed estimation algorithm can accurately estimate the walking speed of older adults.

## Introduction

Human gait is the bipedal, biphasic, forward propulsion of the center of gravity. Because gait is achieved through complex cognitive–motor interactions, [1] it can become abnormal in movement disorders and in cognitive disorders such as Alzheimer’s disease (AD). [2, 3] Furthermore, gait impairment frequently precedes cognitive impairments in cognitive disorders. [4, 5] Among the various gait parameters, walking speed has been studied the most. Moreover, it has been observed to be predictive of a range of outcomes including response to rehabilitation, functional dependence, frailty, mobility disability, cognitive decline, falls, institutionalization, hospitalization, and mortality in both clinical and research settings. [6, 7] Therefore, the White Paper published in 2009 designated walking speed as the sixth vital sign. [6]

In the majority of previous clinical research, walking speed was manually measured using a stop watch over a short walking distance. [7] However, these manual measurements were subject to human error and raters’ subjectivity. Furthermore, the measuring procedures such as walking distance (2–40 m), start (static versus dynamic), path (straight versus turn), and speed (self-selected versus maximal) varied considerably. [6] Laboratory-based motion capture systems and instrumented walkways provided more accurate and instantaneous walking speeds than the manual measurements did. However, these systems are expensive, require trained personnel, and have spatial constraints. These make it infeasible to evaluate walking over sufficient distance and duration and to evaluate natural walking in real-life conditions.

Recently, inertial sensors began to be employed for analyzing gait features including walking speed. Because they are light, wearable, and inexpensive, they can measure gait for longer durations in unsupervised and real-life conditions. However, because inertial sensors do not directly measure walking speed, previous research estimated walking speed using inertial sensors based on one or more of three approaches: kinematic human gait modeling, direct integration, and regression modeling.[8] Apart from the direct integration methods, which tend to drift over time or have constraints of sensor-attachment locations to use zero velocity updates [9–13], or human gait model methods, which are less accurate without calibration and exact measurement[14–18], regression models have emerged as a preferred method of walking speed estimation based on a set of inertial features.[19]

In the regression-based approaches, walking speed is estimated based on input features from the inherent pattern of the inertial measurement unit (IMU) signals and a set of other personal characteristic parameters such as age, gender, height and weight. [20, 21] Because predefined kinematic human gait models are not necessarily required in these approaches, signals from various body parts for which the widely used kinematic human gait model is not established can potentially be used as input to a regression model.[8] This implies that accurate estimation and flexibility of application could be attained by incorporating appropriate data-driven terms representing the gait characteristics of the population. Additionally, the location of the sensor is highly flexible as opposed to that in direct integration (e.g., can be attached to the waist or wrist for long-time monitoring without discomfort), and there is lower disadvantage of drifting over time. However, simultaneously, the performance of the models is highly dependent on the completeness of the training data and the set of parameters in the regression models. To the best of our knowledge, a limited number of studies based on the regression model have been published.

In three of these, the sensor was attached to the lower body [22–24]; in one, the head [25]; and in another, the wrist.[19, 26] Although the studies have revealed highly potential results (mean absolute error = 4.5–5.4%, or concordance correlation = 0.93) for walking speed estimation in the specified dataset, the generalizability would be restricted because the models were derived with training dataset which contained 8–30 subjects (mean age 27–37 years). It is challenging to prevent overfitting with this number. In addition, there is no study that included older adults, although older adults have different gait characteristics from younger adults.[27, 28] Because the slowing of gait is a key feature of older adults and the errors in estimating gait speed increase as the gait speed decreases [9], the algorithm for estimating walking speed in older adults should be designed to reduce errors at low walking speeds.

In this study, we developed a regression-based algorithm for estimating walking speed using IMU-signal data from a large dataset of older adults. In addition, we comprehensively evaluated demographic, anthropometric, and clinical parameters to determine their validity for incorporation in the algorithm. Moreover, we developed the algorithm that can improve the estimation accuracy in the elderly population by additionally applying a model trained specifically in the group with slow walking speed. Finally, the study describes the concurrent validity of the algorithm in comparison with the walking speed measured by an instrumented walkway gold standard, and compares the estimation accuracy of the novel algorithm with those of other algorithms based on the human gait model.

## Materials and methods

### Participants

We enrolled 785 participants from two cohort studies: 495 from the Korean longitudinal study on cognitive aging and dementia (KLOSCAD) [29] and 290 from the Korean frailty and aging cohort study (KFACS).[30] The KLOSCAD and KFACS are population-based prospective multicenter cohort studies conducted in Korea. The KLOSCAD was launched in 2009. Under this study, 6,818 elderly Koreans aged 60 years and over are being followed every two years. The KFACS was launched in 2016. Under this study, 3,000 elderly Koreans aged 70–84 years are being followed every 2 years. Among the 785 participants, 659 (289 men and 370 women) were included in the present analysis after excluding the participants who were pre-frail or frail according to the Korean version of fatigue resistance ambulation illness low weight scale (K-FRAIL) [30], or had obtained 20 points or below in the Tinetti performance oriented mobility assessment (POMA).[31]

All the participants had provided written informed consent themselves or via their legal guardians. This study had been approved by the Institutional Review Board of the Seoul National University Bundang Hospital.

### Measurement of gait

We evaluated parkinsonian symptoms and gait disturbances using the UPDRS (range 0–108, with higher scores indicating more severe parkinsonian motor symptoms) [32] and Tinetti POMA. The Tinetti instrument consists of three scales: a Gait Scale, a Balance Scale, and an overall Gait and Balance score. The maximum score is 28. A higher score represent a higher performance. We also measured the gait of each participant by using a FITMETER^®^ (FitLife Inc., Suwon, Korea,) laced over the center-of-body-mass (CoM) and a GAITRite^™^ (CIR Systems Inc., Havertown, PA) simultaneously. The FITMETER is an IMU and incorporates a digital tri-axial accelerometer (BMA255, BOSCH, Germany) and gyroscope (BMX055, BOSCH, Germany). The sensor is a hexahedron (35 × 35 × 13 mm) with smooth edges and weighing 14 g. It can measure tri-axial acceleration up to ±8g (with resolution 0.004g) and tri-axial angular velocity up to ±1,000°/s (with resolution 0.03°/s). Measurements were made at a sample rate of 250 Hz. The GAITRite is a portable gait analysis walkway system that measures temporal and spatial gait parameters via an electronic walkway connected to the USB port of a computer. Its walkway size is 520 (L) × 90 (W) × 0.6 cm (H), with an active sensing area of 427 (L) × 61 cm (W). It contains 16,128 sensors placed with a spatial accuracy of 1.27 cm. Measurements were made at a sample rate of 100 Hz.

In the present study, we fixed a FITMETER at the level of the third–fourth lumbar vertebrae of each participant by using a Hypafix. Then, we asked each participant to walk back and forth three times on a 14 m flat straight walkway at a comfortable self-selected pace, and to start turning after passing the 14 m line. We placed the GAITRite electronic mat in the middle of the walkway to measure steady state walking. We excluded the 2 m long walks prior to the turns to eliminate the influence of turning. Similarly, the 2 m walk after each start was eliminated.

### Signal preprocessing

**Fig 1:**
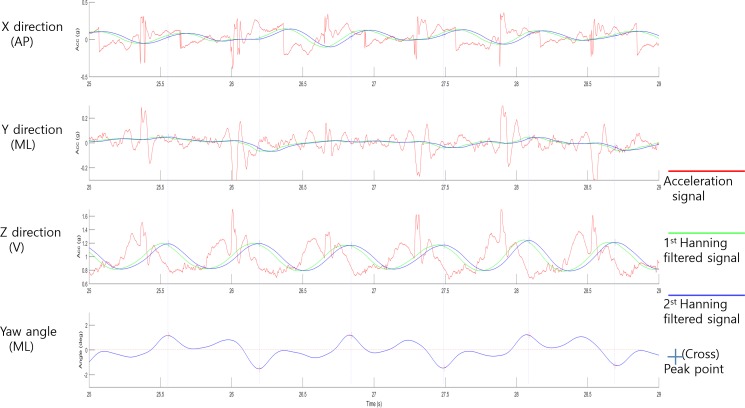
Signal preprocessing. The signal to which the moving average filters (Hanning filters) are applied (green, blue line) shifts to the right compared to the raw acceleration signal, on the time axis. Step discrimination points are indicated by crosses on the filtered signal. Whether the step is left or right is determined by the sign of the yaw angle at the step discrimination point.

Because an IMU attached to each participant had its own local coordinates, a coordinate transform from the local Cartesian coordinate to the global Cartesian coordinate was performed by applying a complementary filter to the raw acceleration and angular velocity data.[33] The anterior–posterior (AP) direction, medio-lateral (ML) direction, and vertical (V) direction were defined as the x-, y-, and z-axes, respectively, of the acceleration data in this study. We applied a low pass filter to the acceleration data in the global Cartesian coordinate to enhance the discrimination rate of step and direction. Two consequential moving average filters (Hanning filters) were applied. The window sizes of the filters were 0.32 s and 0.08 s, respectively, according to the subjects’ average cadence, which is 0.5–0.6 s.[34] We analyzed the data of the central 10 m of the 14 m walking distance to measure steady state walking. Each step was identified from the vertical acceleration data by using the peak detection method. The time gap between consecutive peaks was set to at least 0.24 s. [17] The left and right steps were discriminated using the sign of the yaw angle at the peaks in Fig 1.

### Feature extraction and selection

For each subject, the following demographic, anthropometric, and IMU features were calculated using data from six 10-m-long straight walks. Age and gender are the two demographic features, whereas height, weight, leg length, upper body length, waist circumference, and two feet-lengths of the subjects are the seven anthropomorphic features. The UPDRS and the POMA scores are clinical features reflecting performance of gait and balance. The IMU features include cadence, coefficient of variance of step time (CV step time), magnitude of the 3D acceleration and vertical acceleration, and vertical displacement of CoM within a step cycle. For each step detected in the IMU signal, the magnitude of acceleration and the vertical displacement of CoM were calculated. To do so, we updated the vertical initial velocity at the start of each step, assuming that the vertical heights of CoM and end of a stride were the same in steady state walking, and computed the vertical displacement by integrating the vertical acceleration value during a single step cycle. We used the mean of those values as the features for each subject. The linear relationship between each candidate’s features and walking speed measured with the gold standard was then evaluated using univariate linear regression analysis. We selected the features that could be included in the regression model for walking speed estimation, by considering statistically significant correlation with walking speed and collinearity. We calculated the variance inflation factor (VIF) to evaluate multicollinearity and excluded features with a VIF of 2.5 or higher among the selected features to avoid multi-collinearity according to Allison’s criteria. [2] To do so, the variable with the highest VIF score was dropped until all remaining variables had a score of <2.5. The above process of selecting the suitable features for estimating walking speed was carried out one time each in a whole dataset and a subset group with low gait speeds. This was because features for estimating low speed accurately may be different from those for estimating the whole range of gait speed. Low walking speed was defined as a speed less than 100 cm/s, which is equal to the threshold in a previous study. [9]

Age, gender, cadence, vertical displacement CoM, and foot lengths were the final five features selected using multivariate regression analysis with stepwise selection, from the whole dataset. The features exhibited significant correlation with gait speed and satisfied the condition of collinearity. For the group of subjects exhibiting low walking speeds, age, gender, vertical displacement of CoM, CV step time, and foot length were selected for the regression model. The measurement or computation methods of the selected features in the models are provided in the **Table 1**.

**Table 1:** Features for walking speed estimation.

Feature	Measurement / computation	Whole dataset	Dataset of subjects with slow speed
Demographics			
Age		V	V
Gender		V	
Anthropometries			
Foot length (cm)	Mean of both feet lengths measured by GAITRite	V	V
IMU features			
Cadence (steps/min)	$\frac{60\;(s/min)}{Mean\;Step\;Time\;(s)}$	V	V
Vertical displacement (cm)	Mean (Max–Min of vertical height within a step)	V	V
CV step time (%)	$\frac{SD\;of\;step\;time}{Mean\;Step\;Time}\times 100$		V

Note. IMU: inertial measurement unit; Max: maximum; Min: minimum; CV: coefficient of variance; SD: standard deviation

### Model development and validation

**Fig 2:**
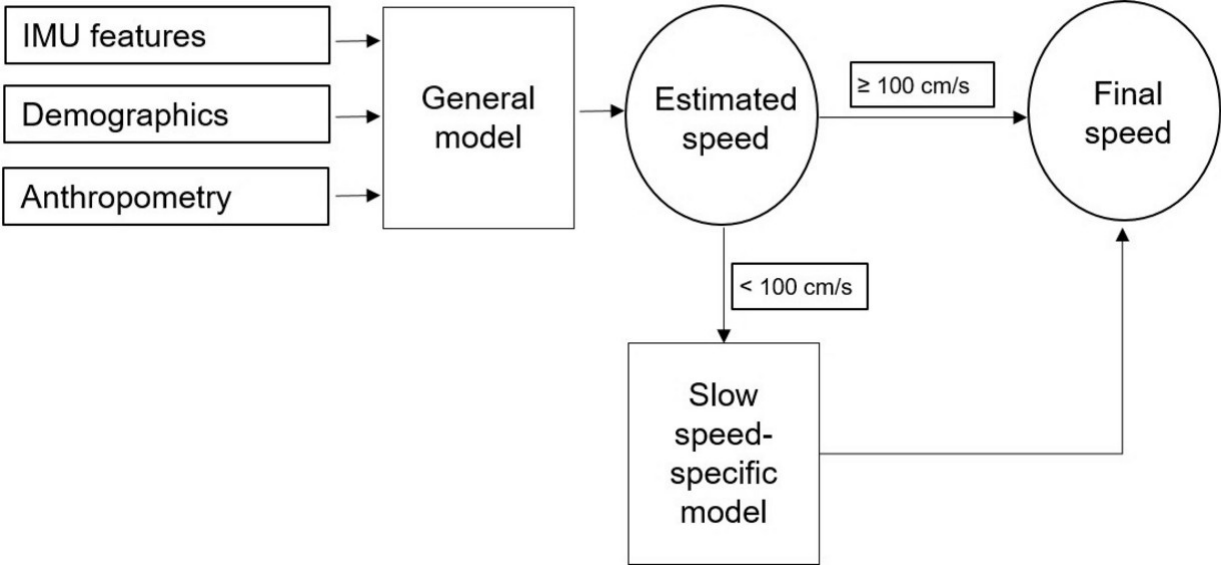
Overview of the walking speed estimation algorithm.

We developed two regression models to be applied sequentially: the general model and the low speed-specific model. To develop the models, we randomly divided the entire dataset into the derivation dataset (70%) and validation set (30%). The general model used five features selected from the whole dataset. The model was fitted using data from the derivation dataset and tested on the validation data. The model derivation and validation processes were carried out in MATLAB (version R2016b) and SPSS v.19.0 (IBM corp., New York, NY). The “regress” function (multiple linear regression using least squares) in MATLAB was applied to obtain the vector of regression coefficients for the linear combination of the features attributed to walking speed. To investigate whether the inclusion of demographics such as gender and age in the estimation model improves accuracy, the models excluding gender, age, or both and the models including these were compared. The final criterion for inclusion in the general model was the minimization of the Akaike information criterion (AIC). The AIC is a likelihood-based measure in which lower values indicate better fit and which extracts a penalty for increasing the number of variables. Thus, the variables selected for inclusion should provide the best fit as well as a parsimonious prediction model. Once the general model was developed, the low speed-specific model was fitted using the low-speed group data (data from individuals whose speeds estimated using the general model were less than 100 cm/s) in the derivation dataset. As in the case of the development of the general model, we compared the AIC values of the model including the five features and the model that excluded gender and selected better-fit model. The final algorithm we propose estimates the walking speed by applying the general model and the low speed-specific model consecutively. First, the general model is applied to estimate the walking speed. If the estimated speed is below 100 cm/s, we applied the low speed-specific model instead of general model to the subject to get more accurate estimation result. (**Fig 2**)

As measures of the accuracy of the walking speed estimation algorithm, the mean absolute error (MAE) and the root mean square error (RMSE) on the validation set were determined. We demonstrated that the algorithm, a combination of the general and low speed-specific models as described in **Fig 2** (referred to as M_1_ hereafter.), exhibits higher performance than the algorithm applying the one-step general model, by comparing the estimation accuracy of the entire validation set with that of its subset with low speed.

### Comparison with other models

The novel regression-based algorithm recommended in this study is denoted as M_1_. M_2_–M_5_ represent four comparative algorithms based on the human gait model. Among the four algorithms, M_2_ and M_3_ are based purely on the human gait model. M_2_, proposed by Zijlstra [17], is based on the inverted pendulum gait model and combines step length with step frequency. M_3_ is its modification obtained by adding the foot length to the term $2\sqrt{2lh-h^{2}}$ in the step-length estimation equation: [35]

$$M_{2}=Cadence∙2\sqrt{2lh-h^{2}}$$

$$M_{3}=Cadence∙(Foot\;length+2\sqrt{2lh-h^{2}})$$

*l=leglength;h=variationsinheightofCoM.

M_4_ and M_5_ are modifications of M_2_ and M_3_, respectively, obtained by multiplying the calibration coefficient C_1_ to the step length term and then adding the intercept constant C_0_ to the product. Because we fitted these models to the linear regression equation using the human gait model term, we refer to the models as fitted human gait models in this paper.

$$M_{4}=C_{0}+C_{1}∙Cadence\sqrt{2lh-h^{2}}$$

$$M_{5}=C_{0}+C_{1}∙Cadence∙(Foot\;length+2\sqrt{2lh-h^{2}})$$

### Statistical analysis

We determined the demographic and anthropometric parameters as means and standard deviations (for continuous variables) or percentages (for the discrete variable). We performed group comparison between the derivation and validation sets, with Chi-square and Fisher’s exact tests for categorical variables and student t-test for continuous variables.

We demonstrated the concurrent validity of each model based on the intraclass correlation coefficients (ICCs) calculated using a two-way mixed model (absolute agreement type) by comparing the walking speeds estimated from each model and those obtained from GAITRite. Additionally, we measured the accuracy of the walking speed estimation models with the MAE and RMSE. The Statistical analysis was performed using SPSS v.19.0.

## Results

**Table 2** presents the subjects’ characteristics in the derivation and validation sets. The subjects have an average age of 73.9 ± 4.7 years (mean ± standard deviation), average weight of 61.9 ± 9.0 kg, average leg length of 84.5 ± 5.6 cm, average feet length of 23.4 ± 1.4 cm, average UPDRS score of 0.8 ± 2.2, average POMA of 27.7 ± 0.8, average walking speed of 113.7 ± 17.9 cm/s, and average cadence of 115.4 ± 9.1 cm/s. The subjects in the derivation and validation sets exhibit comparable clinical characteristics and gait parameters (**Table 2**).

**Table 2:** Baseline clinical characteristics and gait parameters in the model derivation set and the model validation set.

	Derivation set	Validation set	*p*^b^
	(N = 462)	(N = 197)	
Gender (women)	56.9%	54.3%	.594
Age^a^	74.4 ± 5.2	73.6 ± 4.2	.592
Weight (kg)	62.0 ± 9.0	61.7 ± 9.2	.686
Leg length (cm)	84.4 ± 5.5	84.7 ± 6.0	.547
Foot length (cm)	23.4 ± 1.4	23.4 ± 1.4	.997
UPDRS	0.9 ± 2.2	0.6 ± 2.0	.225
POMA	27.7 ± 0.8	27.8 ± 0.6	.104
Speed (cm/s)	113.6 ± 16.8	113.8 ± 20.2	.910
Cadence (steps/min)	115.3 ± 8.9	115.6 ± 9.7	.742
CV step time (%)	3.3 ± 1.5	3.6 ± 3.0	.213

*Note*. UPDRS, Unified Parkinson's Disease Rating Scale; POMA, Performance-Oriented Mobility Assessment; CV step time, Coefficient of Variance of step time.

^a^Data are presented as mean ± standard deviation.

^b^Chi-square test for categorical variable and student t-test for continuous variables

### Model derivation and development

The general model with the smallest AIC value includes five features: age, gender, cadence, vertical displacement CoM, and foot length. The β coefficients, standard errors, standardized beta, t values, and p values of the best-fitting general model are presented in **Table 3**. Age displayed a negative correlation with walking speed. Meanwhile, the female gender, vertical displacement, cadence, and foot length correlated positively with walking speed. The vertical displacement of CoM exhibited the highest standardized beta value (B = 0.582, p < .001), closely followed by cadence (B = 0.581, p < .001). **Table 3** also presents the β coefficients, standard errors, and P values of the features in the low speed-specific model. In the derivation data set, the AIC value for the general model (AIC = 3136.0) was smaller than those of the simplified general models (age-excluded model, AIC = 3157.9; gender-excluded model, AIC = 3143.1; age-and-gender-excluded model, AIC = 3167.0), indicating the increase in predictive capability with the incorporation of the age or gender term. **(S1 Table)**

**Table 3:** General model and low-speed-specific model for walking speed estimation based on data from the model derivation sets.

	General Model (n = 462)			
	β (SE)	B	t	*p*
Gender	2.93 (.970)	.087	3.03	.003
Age	-.365 (.074)	-.100	-4.93	< .001
Cadence	1.09 (.040)	.581	27.6	< .001
Vertical displacement	12.7 (.478)	.582	26.5	< .001
Foot length	3.16 (.331)	.273	9.56	< .001
Constant	-105.5 (12.0)		-8.78	< .001
	Low Speed Model (n = 89)			
	β (SE)	B	t	*p*
Age	-.237 (.149)	-.111	-1.59	.115
Cadence	.874 (0.11)	.628	8.06	< .001
CV Step time	-.520 (0.23)	-.153	-2.23	.028
Vertical displacement	18.8 (1.91)	.713	9.84	< .001
Foot length	.908 (0.55)	.121	1.66	.101
Constant	-50.4 (22.4)		-2.25	.027

*Note*. Body weight is in kg. Leg length, upper body length, and vertical displacement are in cm.

CV, Coefficient of Variance; SE, Standard Error

The low speed-specific model with the smallest AIC value incorporates five features: age, cadence, CV step time, vertical displacement, and foot length. Unlike the general model, the gender term was replaced by the CV step time term. The walking speed exhibited a negative correlation with age and gait variability, and a positive correlation with vertical displacement, cadence, and foot length. The vertical displacement of CoM displayed the highest standardized beta value (B = 0.713, p < .001), followed by cadence (B = 0.628, p < .001) and CV step time (B = -0.153, p = .028). In the derivation data set, the AIC of the low speed model (= 3138.6) was smaller than those of the simplified model (age-excluded model, AIC = 3242.2), indicating the increase in the predictive capability with the incorporation of the age term. **(S1 Table)** The best-fitting general and low speed-specific models are as follows:

#### General model

walking speed (cm/s) = -105.5 + 2.93 × gender (if female) - 0.365 × age + 1.09 × cadence + 12.7 × vertical displacement (cm) + 3.16 × foot length (cm)

#### Slow speed model

walking speed (cm/s) = -50.4–0.237 × age + 0.874 × cadence– 0.520 × CV step time (%) + 18.8 × vertical displacement (cm) + 0.908 × foot length (cm)

### Model testing and comparison with other models

**Fig 3:**
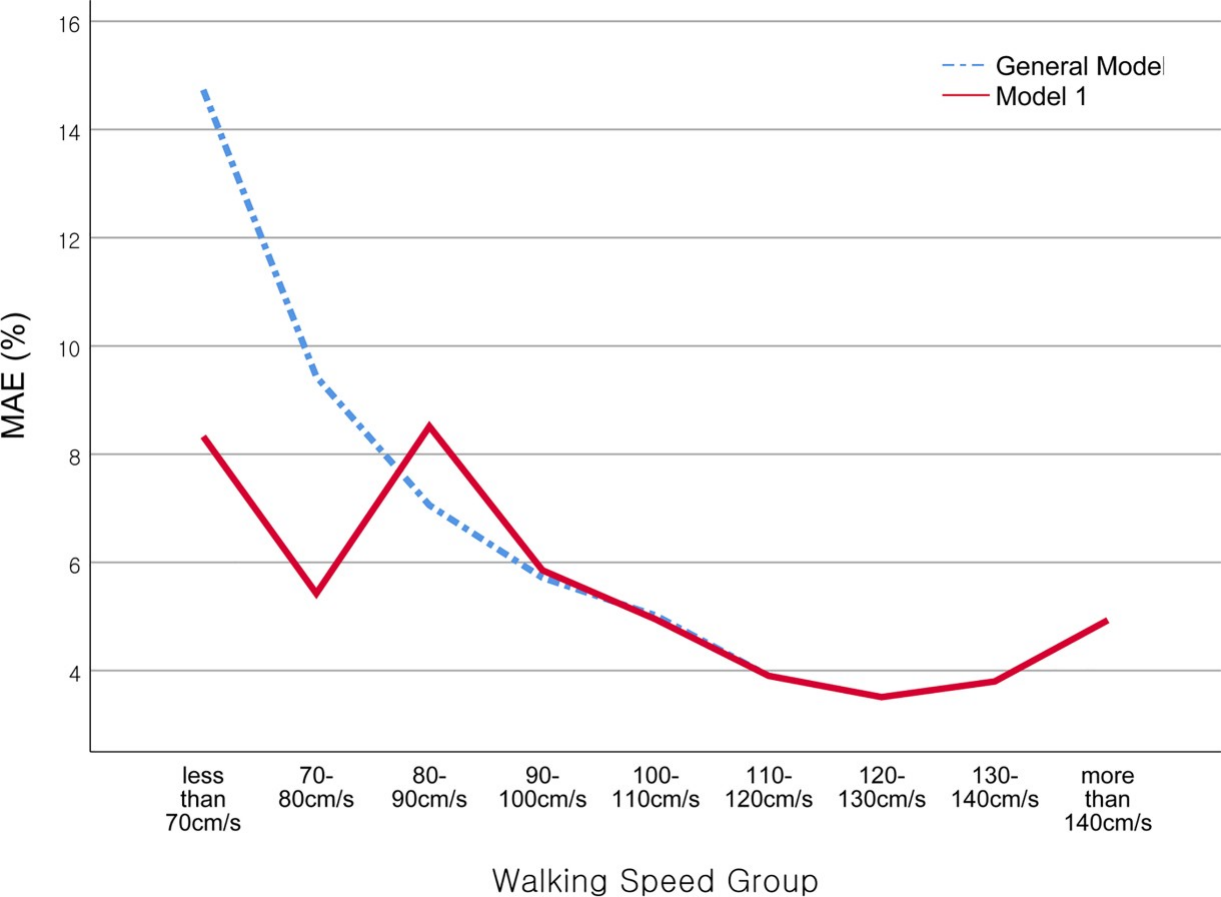
Accuracy of walking speed estimation algorithm according to reference walking-speed group. The general model (indicated by the dotted line) exhibits an inverted j-shaped curve. The MAE increases gradually at low walking speeds. However, the algorithm applying the low-speed-specific model (M1, indicated by the solid lines) limits the MAE increase at low walking speeds within a certain range. (*Note*. MAE, Mean Absolute Error).

**Table 4** presents the accuracy of M_1_ and the general model in terms of walking speed estimation in the 197 older adults (90 men, 107 women) assigned to the validation data set. Although the general model estimated the walking speed with high accuracy, the accuracy varied depending on the walking speed to be estimated. The accuracy for the entire validation set was 4.96% for the MAE and 6.93 cm/s for the RMSE. However, for groups with walking speeds below 100 cm/s (21.3% of the validation dataset), the estimation error was significantly increased by 58% (from 4.96% to 7.84%) in terms of the MAE and 16% (from 6.93 cm/s to 8.01 cm/s) in terms of the RMSE. Moreover, the estimation error increased gradually as the speed decreased (**Fig 3**). The accuracy of slow speed model for the slow speed group was MAE = 6.07%, RMSE = 7.24 𝑐𝑚/𝑠, and concurrent validity (ICC (3,1)) = 0.916. Meanwhile, the newly proposed M_1_ algorithm estimates the walking speed more accurately than the general model does, particularly in the low-speed group. The accuracy for the low-speed group in terms of MAE is 6.69%. This is approximately 1.2% lower than that of the general model. For the entire validation set, M_1_ also provides improvement over the general model. However, the improvement is less apparent than that observed in the low-speed group in the validation set (**Table 4, Fig 3**).

**Table 4:** Comparison of estimation accuracy between model 1^a^ and general model.

	Entire validation set (N = 197)		Slow speed group (N = 42)	
	MAE (%)	RMSE (cm/s)	MAE (%)	RMSE (cm/s)
General model	4.96	6.93	7.84	8.01
Model 1	4.70	6.81	6.69	7.43

*Note*. MAE, Mean Absolute Error; RMSE, Root Mean Square Error.

^a^Model 1 is an algorithm that applies a low-speed-specific regression model sequentially after speed estimation with a general regression model.

**Fig 4:**
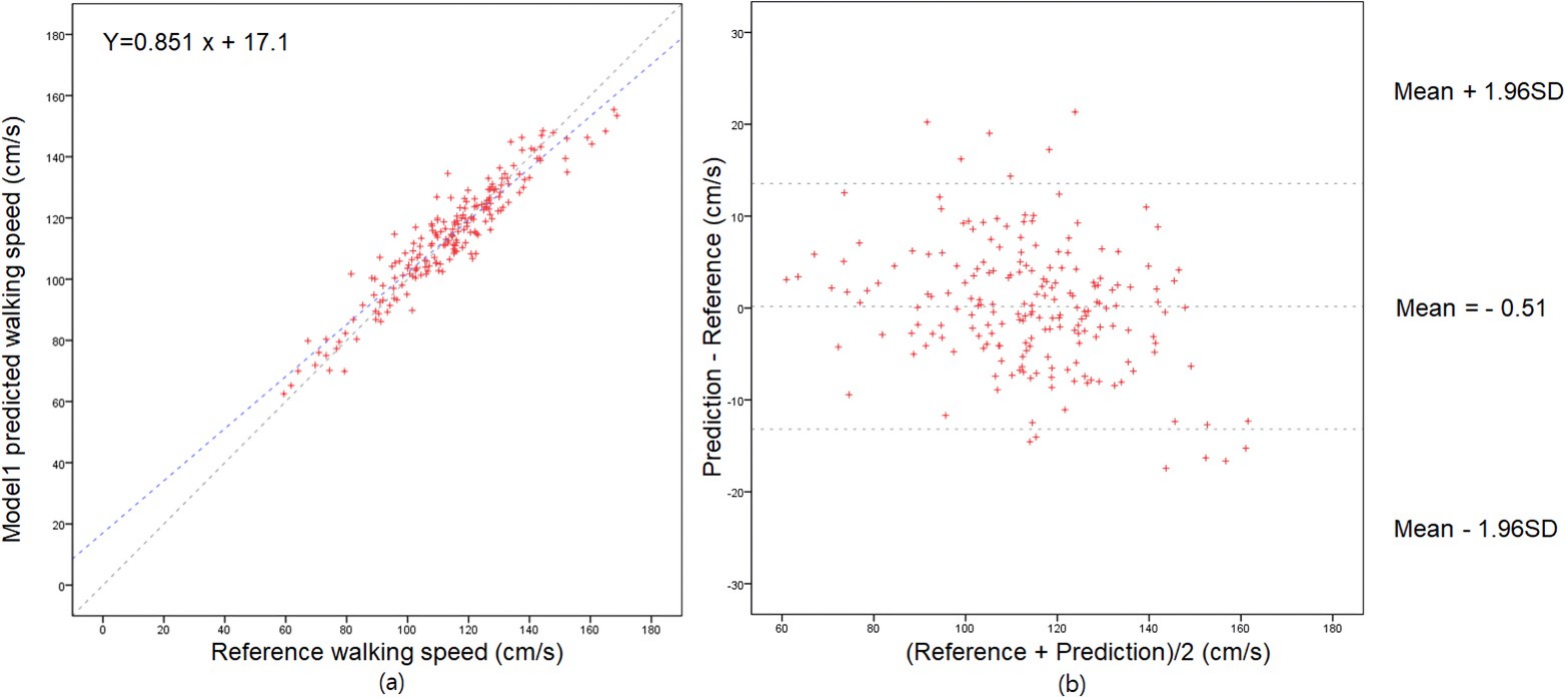
Regression and Bland–Altman Plots. (a) Regression analysis of walking-speed estimation model M_1_ and (b) Bland–Altman plot for comparison of walking speed measured by GAITRite and predicted values from M_1_. The upper and lower horizontal lines represent the 95% limits of agreement, and the middle horizontal line represents the bias.

**Fig 4A and 4B** show the regression analysis and Bland–Altman plot, respectively, for the predicted walking speed based on M_1_. The solid line in **Fig 4A** is the best fit line (y = 0.851x + 17.1). The dotted line is the ideal line (y = x), which represents a perfect correlation between walking speed from GAITRite and walking speed predicted by M_1_. Although the best fit line deviates marginally from the ideal line, the analysis reveals a very strong linear correlation between the predicted and reference walking speeds (Pearson’s r = 0.942, p < .001). The Bland–Altman plot reveals that the error is largely within the 95% limit of agreement even in the range of speeds less than 100 cm/s.

**Table 5** presents summary statistics regarding the performance of M_1_ and its four comparative models (M_2_–M_5_) in terms of walking-speed estimation. For each pre-specified summary statistic (MAE, RMSE, or ICC), M_1_ provided improvement in accuracy over M_2_ and M_3_, the prediction algorithms based on the human gait model (MAE of 4.90% vs 20.9% and 19.5%, respectively; RMSE of 6.81 cm/s vs 26.0 cm/s and 22.1 cm/s, respectively). It also exhibited remarkable concurrent validity with GAITRite (ICC of 0.937 with 95% confidence interval [0.918 0.952]). The algorithms purely based on the human gait model exhibited concurrent validities with the GAITRite at the low-to-moderate level (ICC of 0.446 for M_2_ and 0.585 for M_3_). The mean absolute error was over 20%, and the RMSE exceeded one standard deviation of walking speed. The fitted human gait models have considerably higher accuracy. The MAE and RMSE of M_4_ were 6.25% and 8.63 cm/s, respectively, approximately one-third of those of M_2_. Moreover, the concurrent validity was remarkable. The accuracy of the M_5_, which incorporates the foot length term, was higher than that of M_4_ and lower than that of M_1_. M_1_ exhibited the highest accuracy and concurrent validity among the five models (**Table 5**).

**Table 5:** Evaluation of walking speed estimation accuracy.

	Validation set (n = 197)		
Model	ICC (3,1)^a^ [95% CI]	MAE (%)	RMSE (cm/s)
*Regression models*			
Model 1	0.937 [0.918 0.952]	4.70	6.81
*Human gait models*			
Model 2	0.446 [-0.058 0.783]	20.93	25.99
Model 3	0.585 [-0.052 0.865]	19.47	22.07
*Human gait models (Fitted)*			
Model 4	0.893 [0.861 0.918]	6.24	8.63
Model 5	0.920 [0.895 0.939]	5.44	7.59

*Note*. ICC, Intraclass Correlation Coefficient; CI, Confidence Interval; MAE, Mean Absolute Error; RMSE, Root Mean Square Error; SD, Standard Deviation.

^a^Intraclass Correlation Coefficients are presented with 95% confidence interval.

## Discussion

In this study of 659 Korean older adults, we developed and validated an algorithm for walking speed estimation based on the regression method. The algorithm consists of variables such as age, gender, and foot length, which are already known to the individual and readily available, as well as vertical displacement of CoM, cadence, and CV step time, which were obtained from an IMU sensor. It exhibited high accuracy in estimating the walking speed of the older adults and a remarkable concurrent validity compared to the gold standard. Although the estimation error, in general, increased gradually as the speed decreased, the algorithm we propose achieved high estimation accuracy even at the low walking speed by applying a low speed-specific regression model, sequentially after estimation by a general regression model. The newly developed algorithm exhibited a substantially higher accuracy when compared with models based on the human gait model without calibration to fit the population. This high accuracy was verified in a completely exclusive validation dataset of a sufficiently large size.

We demonstrated that the regression-based algorithms proposed in this study exhibit substantially higher estimation accuracy than pure-model-based algorithms. This is consistent with the previous study in that model-based methods require subject- or population-specific model calibration, which is a disadvantage of the method.[36] The fitted human gait models that completed the population-specific calibration exhibited substantially higher accuracy than the pure-model-based algorithms. However, their accuracy was less than that of the regression-based algorithm M_1_. In addition to the substantially higher accuracy, the novel algorithm circumvents the use of the leg length term included in the gait-model-based algorithms. This is another advantage because if anthropometric measurement is required for each individual, it will be inconvenient in that each individual needs to visit an institution or consult trained personnel for reliable leg length measurement, prior to the application of the model to the individuals.

The accuracy measure values obtained from the linear regression model proposed in this study are MAE = 4.70% and RMSE = 6.81 𝑐𝑚/𝑠. Moreover, the concurrent validity is ICC (3,1) = 0.937 in the older adult population. These are comparable to the results of previous regression-based methods (MAE 4.5–5.4% or concordance correlation of 0.93) in the young adult population.[19, 23, 24] Zihajehzadeh et al. developed and validated the Gaussian process regression (GPR) model for walking speed estimation with accuracy of MAE = 4.50% and RMSE = 5.30 𝑐𝑚/𝑠 in the young healthy adult population (average age = 27 ± 4 years). Recently, Soltani et al. developed an algorithm that estimates step length to less than 5% of MAE using machine learning (regression-based) in 30 healthy adults. It is noteworthy that even a simple linear regression model comprising age, gender, foot length, and three gait features from an IMU proposed in this study exhibits a high accuracy of estimating walking speed in older adults. Both the low-speed-specific model and the general model incorporated the age term in the best-fitting speed estimation equations. Although the general model includes the age term as well as the gender, the age term plays a more important role in speed estimating because the AIC reduction is substantially larger when the age item is incorporated. The negative beta value of the age term indicates that as age increases, the speed estimated from the IMU features and anthropometrics should be adjusted downward. The observations indicate that to estimate gait speed more accurately, the age term is required in addition to the terms used in the inverted pendulum model, such as vertical displacement, cadence, and foot length. This may imply that the human gait is not accurately modeled by the inverted pendulum model, with ageing.

It is also an important observation that notwithstanding the remarkable overall performance of the general model in the older adults, the estimation accuracy of the model is considerably lower at walking speeds less than 100 cm/s. This is consistent with a previous study regarding regression-model-based walking speed estimation using a wrist-worn inertial sensor.[9] The accuracy in the group with walking speeds of 50–100 cm/s was RMSE = 7.5 cm/s and MAE = 8.9% in the previous study, and RMSE = 8.01 cm/s and MAE = 7.84% in the present study. The lower performance of the regression model at low walking speeds should be considered particularly while estimating walking speed in the elderly by using a regression model with the IMU sensor features. The walking speed of 100 cm/s, used as the threshold value of low walking speed in this study, was widely used as the threshold for frailty or sarcopenia in previous studies.[37, 38] In a previous study, 16.8% belonged to the low gait speed (less than 100 cm/s) group.[38] Meanwhile, in our study, 19.9%, or one in five, belonged to the low gait speed group in the elderly population. In older adults, various pathological conditions such as stoke, Parkinsonism, excessive white matter hyperintensity, cardiovascular disease, and spinal stenosis, as well as frailty and sarcopenia are more prevalent than in the younger population. These conditions cause a slowing of gait.[39] Moreover, such pathological conditions may cause variation in gait patterns during walking, hindering the estimation of the walking speed using a generalized regression-based model. Our observations demonstrate that a low-speed-specific model can reduce the MAE from 7.84% to 6.69% in the low walking speed groups. In addition, the MAE difference between the general model and the low-speed-specific model tended to increase with lowering of speed, within the low walking speed group. This may cause the difference between the two models to increase in a population with a high proportion of elderly with low walking speeds. Therefore, we recommend that an algorithm implementing a specific model that reflects the characteristics of groups with low walking speeds be used for older adults. We consider this to be even more vital in the hospitalized or institutionalized elderly population than in the community-dwelling elderly population, the target population of the present study.

Our algorithm exhibited slightly lower but comparable accuracy to the previous speed estimation algorithms using waist or pelvis worn single IMU. [11, 40] An algorithm using the kinematic human gait model showed accuracy of 3.36% for MAE and 3% for MAE in studies using direct integration. The previously proposed algorithms were validated in healthy young adults, and the difference in study population with our study may explain the small difference in accuracy. Even for the older adults, however, there were a few studies on the direct integration-based walking speed estimation algorithm [15, 41] and the accuracies reported in these studies were 1.4%, and 3%, which were higher than ours was. The studies used a pair of IMUs attached to ankles [41] and feet [15], respectively. Since the farther from the contact point the IMU is placed, the more difficult the gait events identification is due to the disadvantage of zero velocity updates [41], the difference in accuracy between previous studies and ours could be explained in part by differences in sensor attachment sites as well as in estimation method. Considering that gait measurement using the IMU is based on the advantage of monitoring for longer durations in unsupervised and real-life conditions at lower cost, it should be taken into account that the waist-placement causes less constraint in body movement and minimal discomfort [42] and using a single sensor rather than a pair of sensors demands lower costs.

A strength of this study is the development of the algorithm from a sufficient number of older adults and verification of the accuracy of the algorithm by applying it on an independent validation set. Another strength of this study is that a low-speed model is independently developed for speed estimation and that the estimation accuracy is improved by applying it as the next step after the application of the general model. However, there are certain limitations. We used an independent validation dataset to verify whether the proposed algorithm is over-fitted to the specific dataset. Nonetheless, it is necessary to apply it to other populations to verify its generalizability. We used the features selected based on the whole dataset in general model. It may cause biased evaluation for fitted model trained by derivation dataset. Nevertheless, the bias might be small because the whole dataset and derivation dataset were homogeneous groups with similar clinical and gait characteristics. In addition, an algorithm for estimating the walking speed of the elderly with speeds of less than 100 cm/s based on the IMU sensor needs to be further studied and improved. In the elderly with walking speeds less than 100 cm/s, additional terms may be required in the fine-tuned regression model in order to reflect divergent gait patterns. In the present study, interaction terms between features were not selected as the features because of the multicollinearity with the raw feature terms in the feature selection stage, but including the interaction terms in the regression model could potentially improve the accuracy of the model. Feature selection methods that are more contemporary such as Least Angle Regression (LARS) or Elastic Net Regression, or models other than linear regression may be required to improve performance of the algorithm. Considering the flexibility to develop a better-fit model by incorporating an appropriate term that reflects the gait characteristics without constraints on the sensor-attachment site, the regression-based model can be effective for measuring the gait in the elderly. Future studies are warranted to develop regression-based models with improved performance, especially in older adults with slow walking speeds.

## Supporting information

S1 Table AIC, and Delta AIC for the different regression models. (DOCX)
