# Supplementary material for: Walking-speed estimation using a single inertial measurement unit for the older adults
Source: PLoS One. 2019 Dec 26;14(12):e0227075. doi: 10.1371/journal.pone.0227075 (PMC6932800; doi:10.1371/journal.pone.0227075)
Supplement: S1 Table — (DOCX) [file pone.0227075.s001.docx]

**Supporting Information**

**S1 Table. AIC, and Delta AIC for the different regression models.**

| **Models** | | **AIC** | **delta AIC** |
| --- | --- | --- | --- |
| **General Model (M*_G_*)** | | 3136.0 | minAIC |
|  | Gender + Age + Cadence + Vertical displacement + Foot length |  |  |
| **M*_G_* - Age** | | 3143.1 | 7.1 |
| **M*_G_* - Gender** | | 3157.9 | 21.9 |
| **M*_G_* - Age - Gender** | | 3167.0 | 31.0 |
|  | |  |  |
| **Slow Speed Model (M*_S_*)** | | 3138.6 | minAIC |
|  | Age + Cadence + CV Step Time + Vertical displacement + Foot length |  | 103.6 |
| **M*_S_* - Age** | | 3242.2 |  |
